# Vitamin K-dependent proteins GAS6 and Protein S and TAM receptors in patients of systemic lupus erythematosus: correlation with common genetic variants and disease activity

**DOI:** 10.1186/ar4199

**Published:** 2013-03-12

**Authors:** Pedro Recarte-Pelz, Dolors Tàssies, Gerard Espinosa, Begoña Hurtado, Núria Sala, Ricard Cervera, Joan Carles Reverter, Pablo García de Frutos

**Affiliations:** 1 Department of Cell Death and Proliferation, Institute of Biomedical Research of Barcelona (IIBB-CSIC, IDIBAPS), C/ Roselló 161 6°, Barcelona, 08036, Spain; 2 Department of Hemotherapy and Hemostasis, Institut d'Investigacions Biomèdiques August Pi i Sunyer (IDIBAPS), Hospital Clinic, University of Barcelona, C/ Villarroel 170, Barcelona, 08036, Spain; 3 Department of Autoimmune Diseases, Institut Clínic de Medicina i Dermatologia, Hospital Clinic, C/ Villarroel 170, Barcelona, 08036, Spain; 4 Translational Research Laboratory, Institut Català d'Oncologia (IDIBELL-ICO), Gran Via, km 2.7 s/n, L'Hospitalet de Llobregat, 08907, Spain

## Abstract

**Introduction:**

Growth arrest-specific gene 6 protein (GAS6) and protein S (ProS) are vitamin K-dependent proteins present in plasma with important regulatory functions in systems of response and repair to damage. They interact with receptor tyrosine kinases of the Tyro3, Axl and MerTK receptor tyrosine kinase (TAM) family, involved in apoptotic cell clearance (efferocytosis) and regulation of the innate immunity. TAM-deficient mice show spontaneous lupus-like symptoms. Here we tested the genetic profile and plasma levels of components of the system in patients with systemic lupus erythematosus (SLE), and compare them with a control healthy population.

**Methods:**

Fifty SLE patients and 50 healthy controls with matched age, gender and from the same geographic area were compared. Genetic analysis was performed in *GAS6 *and the TAM receptor genes on SNPs previously identified. The concentrations of *GAS6*, total and free ProS, and the soluble forms of the three TAM receptors (sAxl, sMerTK and sTyro3) were measured in plasma from these samples.

**Results:**

Plasma concentrations of GAS6 were higher and, total and free ProS were lower in the SLE patients compared to controls, even when patients on oral anticoagulant treatment were discarded. Those parameters correlated with SLE disease activity index (SLEDAI) score, GAS6 being higher in the most severe cases, while free and total ProS were lower. All 3 soluble receptors increased its concentration in plasma of lupus patients.

**Conclusions:**

The present study highlights that the GAS6/ProS-TAM system correlates in several ways with disease activity in SLE. We show here that this correlation is affected by common polymorphisms in the genes of the system. These findings underscore the importance of mechanism of regulatory control of innate immunity in the pathology of SLE.

## Introduction

Systemic lupus erythematosus (SLE) is a chronic autoimmune disease characterized by an impairment of the regulation of the immune system [1]. This is reflected in hyperactivity of lymphocytes, the production of pathogenic auto-antibodies, and the formation of immune complexes, which can lead to multi-organ damage. Although the exact aetiology is not known, one of the possible triggers of this autoimmune reaction is a deficit in the process of efferocytosis, the clearance of apoptotic cells [2]. Under normal circumstances, apoptotic cells are engulfed by macrophages and other phagocyting cells using specific mechanisms, different from those of phagocyting bacteria and other corpuscles [3]. This provides a clearance of possible auto-antigens in the early phase of cell death without inducing inflammation or the immune response. In SLE, however, the clearance of apoptotic cells by macrophages is impaired, which may allow apoptotic cells to serve as immunogens for the induction of autoreactive T and B cells and drive the production of auto-antibodies [4].

The reasons for the defective clearance of apoptotic cells in SLE are not clear. The past decade has provided significant evidence that the complement cascade, immunoglobulin (Ig) M or pentraxins as serum amyloid P-component and C-reactive protein, contribute to the clearance of apoptotic bodies. Macrophages recognize apoptotic cells through an array of surface receptors [3]. Among them, the Tyro3, Axl and MerTK (TAM) tyrosine kinases play an especially important role in the clearance of apoptotic cells by macrophages and dendritic cells [5]. Mice lacking the three TAM receptors rapidly develop lupus-like symptoms, being the MerTK receptor the most potent mediator in this instance [6-8]. The main ligands that bind to and activate the TAM family of receptors are growth arrest-specific 6 (GAS6) and protein S (ProS), which are also capable of binding to negatively charged residues exposed early in apoptosis on the membrane surface of the apoptotic cell [9].

GAS6 and ProS are 75 kDa multimodular vitamin K-dependent glycoproteins (VKDPs) found in chordates, sharing a 40 to 43% amino acid identity in different species. Their modular architecture is identical, containing an N-terminal γ-carboxyglutamic acid (Gla) domain, interacting with phosphatidylserine containing membranes, followed by four epidermal growth factor-like domains and two laminin globular-like domains that contain the interaction site with TAM receptor tyrosine kinases, the first one having a crucial role [10,11]. GAS6 is expressed in many tissues, including capillary endothelial cells, vascular smooth muscle cells, and bone marrow cells. Unlike other VKDPs, GAS6 production in the liver is minor, and its concentration in plasma is approximately 1,000-fold lower than that of ProS [12]. ProS has a critical function in regulating coagulation in several critical steps [13]. ProS circulates as approximately 40% free ProS and 60% as a complex with C4b-binding protein (C4BP); only free ProS is active as a cofactor for activated protein C and a ligand for the TAM receptor kinases [14]. Lower free ProS concentration in plasma is associated with an increased risk of deep venous thromboembolism [15].

TAM receptors undergo a proteolytic processing in the cell membrane, leading to plasma circulating levels of extracellular fragments of the receptors (soluble receptors). These forms have been shown to interact with the ligands, modifying their function [16,17]. These soluble fragments could have regulatory functions for the GAS6/ProS-TAM system, as well as being markers of its state of activation.

Recently, several studies have focused on the components of this system in groups of patients with lupus [18-20]. Low values of ProS might have important roles in the pathogenesis of SLE, and acquired ProS deficiency has been associated with the thrombotic complications often encountered in these patients [21]. In contrast, plasma GAS6 concentration was reported to be elevated in patients with severe sepsis, and septic shock, or inflammatory diseases without infectious agents and severe acute pancreatitis. GAS6 concentration in SLE has been evaluated by two studies, demonstrating either an increased concentration or similar levels in patients with lupus [18,22]. A genetic association of single nucleotide polymorphic variants in the genes of the GAS6/ProS-TAM system has been determined in cardiovascular disease [23-25]. In the present study we correlate the plasma concentration of components of the system with single nucleotide polymorphisms (SNPs) characterized in the GAS6/TAM genes.

## Materials and methods

### Individuals enrolled in the study

Plasma samples from a group of patients with SLE (n = 50) were obtained from the Department of Autoimmune Diseases Hospital Clínic, University of Barcelona, Barcelona, Spain. A group of healthy individuals (n = 50) without autoimmune disease, bleeding disorders, thrombosis or a history of pregnancy loss was obtained from the Department of Haemostasia and Haemotherapy. The two groups were matched for ethnicity (all Caucasians), age (mean age 40 years) and gender (two men and forty-eight women). Patients with SLE fulfilled the revised criteria of the American College of Rheumatology for the classification of SLE [26]. The main SLE clinical manifestations evaluated in this study were defined according to the American Rheumatism Association glossary committee [27]. Disease activity was scored using the Systemic Lupus Erythematosus Disease Activity Index (SLEDAI) [28], while the SLE-associated injury was measured by the Systemic Lupus International Collaborating Clinics/ACR (SLICC/ACR) Damage Index [29]. The present study was approved by the Human Experimental Committee of the Hospital Clínic (Barcelona) and was performed according to the principles of the Declaration of Helsinki. Informed consent was obtained from all participants.

### Parameter determination in plasma samples

Free and total ProS were determined using standard clinical laboratory methods (Asserachrom Protein S and Asserachrom Free Protein S, Stago, Asnières, France). Values are expressed as percentage of control plasma. The concentration of the vitamin K-dependent ligand GAS6, and the extracellular fractions of the receptors, sTyro3, sAxl and sMerTK were analyzed in plasma from controls and patients by ELISA. For determination of GAS6, the technique described by Alciato *et al. *was used, with some modifications [30]. Capture and detection polyclonal antibodies were obtained from R&D Systems. The plates were blocked for 1 hour at room temperature with diluent buffer (PBS with 1% bovine serum albumin, pH = 7.4). The plates were washed three times with wash buffer (PBS with 0.05% Tween20). For the determination of sTyro3 and sMerTK in plasma, ELISA was performed using previously matched capture and detection antibodies (Duo Set reagents from R&D Systems, Minneapolis, MN, USA). Small modifications were introduced in the protocols of the manufacturer. For sMerTK determination the diluent reagent contained 3% fish gelatin instead of albumin, as indicated by Wu *et al. *[20]. For the determination of sAxl, a polyclonal antibody from R&D systems was used as capture antibody, while a biotinylated antibody was used as detection reagent. Dilutions of plasma samples were adapted for each assay. Standard curves were made by serial dilution of purified proteins in each case. Negative controls were buffer alone and a plasma pool was used as the internal standard of the assays.

### Genotype determination and analysis

Genomic DNA was extracted from venous blood samples. Genotype determinations were performed as previously described, using quantitative real-time PCR [23]. In *GAS6 *[EMBL: ENSG00000183087], we selected rs8191973, rs7331124, rs7323932 and rs8191974 SNPs, which form the haplotype block previously associated with stroke. In TAM genes, after a search in the public databases dbSNP, Ensembl and HapMap, we selected eleven SNPs: four in *TYRO3 *[EMBL: ENSG00000092445], three in *AXL *[EMBL: ENSG00000167601] and four in *MERTK *[EMBL: ENSG00000153208]. Inclusion criteria were: to include both validated SNPs with minor allele frequency (MAF) > 0.05, which were also potentially functional SNPs (missense, located in putative splice-site sequences or in both 5'-UP and 3'-DS regions) and/or were SNPs that tagged common haplotypes (minor haplotype frequency of 0.05), according to Haploview analysis of HapMap information on these genes. Genotyping was performed by quantitative real-time PCR. SNPs rs8191973, rs7331124, rs7323932, and rs8191974 in *GAS6 *and rs869016 in *MERTK*, were genotyped using FRET probes in a LightCycler^®^ 480 System (Roche Applied Science, Penzberg, Germany). Amplification primers and probes were designed and synthesized by TIBMOLBIOL (Berlin, Germany). Genotypes of rs2277537, rs16971872, rs2289743, rs12259 in *TYRO3*, rs4802113, rs2304234, rs2304232 in *AXL *and rs10496440, rs7573344, rs17835605 in *MERTK *were genotyped by TaqMan probes in an ABI 7900HT instrument (Applied Biosystems, Paisley, UK), following manufacturer's recommendations. SNP association analysis was performed as reported previously using the SPSS package and SNPStats web tool. Detailed protocols and information on the SNP selection criteria are provided in Hurtado *et al. *[23].

### Statistical analysis

Statistical Analysis was performed with Graphpad Prism v 4.02 (Graphpad Software Inc, La Jolla, CA, USA). Continuous results are expressed as mean ± SD, unless indicated otherwise. Comparisons were made with Student's *t-*test for equal variances or the Mann-Whitney test if variances were different. As treatment with oral anticoagulation affects ProS and Gas6 [12,31], we performed comparisons between SLE patients and controls in the whole groups and after excluding patients who were under oral anticoagulation treatment. Comparisons among plasma parameters and SLEDAI were performed using the Kruskal-Wallis test. Correlation among plasma parameters was evaluated with Pearson's linear regression. *P*-values lower than 0.05 were considered statistically significant. The numbers of each genotype observed were compared with those expected for a population in Hardy-Weinberg equilibrium with the chi-squared test.

## Results

### Patients

Fifty patients with SLE were analyzed. The main demographic and clinical characteristics are depicted in Table 1. Of note, five out of twenty-eight patients (18%) with lupus nephritis developed renal failure, defined as glomerular filtration rate < 60 mL/minute per 1.73 m^2^. In addition, five patients (10%) were on oral anticoagulant therapy.

**Table 1:** Demographic and clinical characteristics of patients with systemic lupus erythematosus (SLE) and healthy controls

	SLE patients (n = 50)	Healthy controls (n = 50)
Demographic characteristics		
Age at study, years, mean ± SD	40.2 ± 10.5	40.3 ± 10.6
Sex, female:male	48:2	48:2
Clinical characteristics		
Arthritis, n (%)	49 (98%)	
Skin involvement, n (%)	41 (82%)	
Glomerulonephritis (class), n (%)	28 (56%)	
	Class IV: 21	
	Class V: 3	
	Class III: 2	
	Class II: 2	
Haematologic involvement, n (%)	21 (42%)	
	Hemolytic anemia 22%	
	Thrombocytopenia 34%	
Serositis, n (%)	14 (28%)	
Neurological involvement, n (%)	8 (16%)	
SLEDAI score, mean ± SD (range)	5.92 ± 5.89 (0, 26)	
SLICC score mean ± SD (range)	1.52 ± 1.59 (0, 7)	
Oral anticoagulation, n	5	0

SLEDAI, Systemic Lupus Erythematosus Disease Activity Index; SLICC, Systemic Lupus International Collaborating Clinics; n, number of patients or controls.

### Plasma values of GAS6/ProS-TAM system parameters in SLE and controls

**Figure 1:**
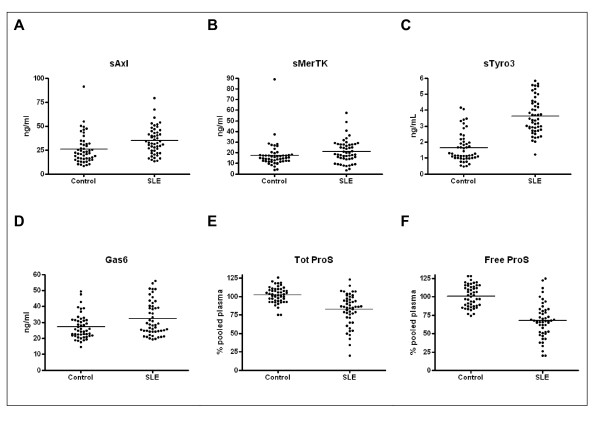
**Plasma concentrations of the components of the growth arrest-specific gene 6 protein (GAS6)/Protein S (ProS)-Tyro3, Axl and MerTK receptor tyrosine kinase (TAM) system in systemic lupus erythematosus (SLE) and in normal controls**. Normal controls were matched for age, ethnicity and geographical area (n = 50). Values were significantly different (*P *< 0.05).

The different protein components of the GAS6/ProS-TAM system were measured in the samples of 50 SLE patients and 50 age- and sex-matched controls (Table 2). The three soluble forms of the TAM receptors were increased in the 50 patients of SLE compared with age- and sex-matched controls (Figure 1a-c). Soluble Tyro3 had the largest increase in concentration, more than double the concentration of the control group (1.65 ± 0.93 ng/mL in controls versus 3.62 ± 1.13 ng/mL in SLE patients), followed by sAxl (1.3-fold increase), and sMerTK (1.2-fold increase).

**Table 2:** Plasma values of the GAS6/ProS-TAM system in patients with systemic lupus erythematosus (SLE) and healthy controls

	SLE patients (n = 50)	Healthy controls (n = 50)	*P*
sAxl, ng/mL	34.8 ± 14.3	25.9 ± 15.3	0.0036
sMerTK, ng/mL	21.2 ± 10.8	17.4 ± 12.1	0.0122
sTyro3, ng/mL	3.62 ± 1.13	1.65 ± 0.93	< 0.0001*
GAS6, ng/mL	32.3 ± 1.50	27.2 ± 1.08	0.0067
ProS, total, %	82.6 ± 3.03	102.4 ± 1.52	< 0.0001
ProS, free, %	67.6 ± 3.34	101.0 ± 2.07	< 0.0001*

Quantitative variables as expressed as mean ± standard deviation. *Significant after Bonferroni correction for multiple comparisons.

In contrast to the receptors, the ligands followed opposite behaviours (Figure 1d-f); while GAS6 values increased 1.2-fold compared to controls, total and free ProS decreased in SLE patients. The decrease was particularly important in free ProS, at more than 30% of the control values. Comparisons were also made after excluding the five patients on anticoagulant therapy, as ProS and GAS6 concentrations are affected by it [12,31], and there were similar significant differences (GAS6 32.2 ± 10.3 ng/mL, *P *= 0.008; total ProS 87.4 ± 16.0% of control plasma; free ProS 71.7 ± 21.0% of control plasma, *P *< 0.0001in both cases). After applying the Bonferroni correction for multiple comparisons in the plasma measurements, sTyro3 and free ProS concentrations remained significantly higher in the SLE group compared to controls.

### Disease activity, severity and plasma values of GAS6/ProS-TAM system parameters

**Figure 2:**
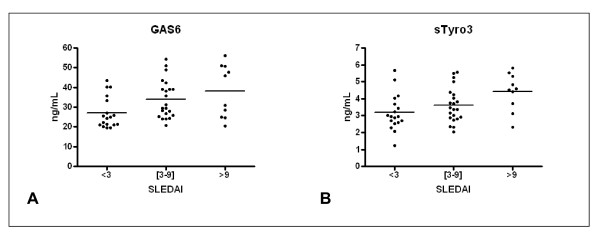
**Correlation of growth arrest-specific gene 6 protein (GAS6) and sTyro3 with Systemic Lupus Erythematosus Disease Activity Index (SLEDAI)**. Systemic lupus erythematosus (SLE) patients were classified according to SLEDAI into a low (n = 18), intermediate (n = 22) or high activity group (n = 10). Values were significantly different using analysis of variance (ANOVA) (GAS6, *P *= 0.015; sTyro3, *P *= 0.021).

Several measures of the GAS6/ProS-TAM system correlated with the severity of the disease (Figure 2). For instance, when we compared the group of patients with a SLEDAI score lower than 3 (n = 18) with those patients with a higher SLEDAI score (n = 32), there was a significant difference in GAS6 plasma concentration (*P *= 0.005), with 27.0 ± 1.9 ng/mL in the low-score group and 35.3 ± 1.9 ng/mL in the high score group (Figure 2A). Indeed, the low SLEDAI-score group had the same GAS6 plasma concentration as the control group. This difference remained significant when the five patients on oral anticoagulants were excluded (*P *= 0.003). In contrast, the TAM receptors had only moderate increases in the high SLEDAI group for sTyro3 (3.2 ± 1.0 ng/mL vs 3.9 ± 1.1 ng/mL; *P *= 0.03) (Figure 2B); or no differences for sAxl (31.3 ± 12.0 ng/mL vs 36.8 ± 15.3 ng/mL; *P *= 0.26) and sMerTK (19.2 ± 10.3 ng/mL vs 22.3 ± 11.0 ng/mL; *P *= 0.29) (data not shown). ProS parameters were also similar in high and low SLEDAI groups when the five patients who were receiving oral anticoagulation were excluded from the analysis (total ProS 84.6 ± 3.4% vs 91.6 ± 3.0%; *P *= 0.13; free ProS 71.2 ± 4.5% vs 72.4 ± 4.2%; *P *= 0.8).

GAS6, sMerTK, and sTyro3 showed a significant positive linear correlation with SLEDAI, but not sAxl (Table 3). While total ProS showed a negative significant correlation with SLEDAI, free ProS values showed no significant correlation. In contrast, no parameter correlated with the SLE-associated injury score, as measured by SLICC, with sMerTK being marginally significant (Table 3).

**Table 3:** Linear correlations of plasma values with SLEDAI and SLICC scores

	SLEDAI		SLICC	
	
	Pearson's *r*	*P*	Pearson's *r*	*P*
GAS6	0.327	0.021	0.187	0.193
Total ProS	-0.359	0.01	0.050	0.741
Free ProS	-0.233	0.103	0.052	0.735
sAxl	-0.008	0.957	0.185	0.198
sMerTK	0.425	0.002	0.285	0.045
sTyro3	0.437	0.001	0.118	0.414

SLEDAI, Systemic Lupus Erythematosus Disease Activity Index; SLICC, Systemic Lupus International Collaborating Clinics/American College of Rheumatology; GAS6, growth arrest-specific gene 6 protein.

### Differences in GAS6/ProS-TAM in specific SLEDAI manifestations

Patients with renal failure (n = 5) presented higher values of GAS6 (42.5 ± 4.0 ng/mL vs 31.2 ± 1.5 ng/mL; *P *= 0.023); MerTK (31.4 ± 7.3 ng/mL vs 20.1 ± 1.4 ng/mL; *P *= 0.024); and Tyro3 (4.6 ± 0.4 ng/mL vs 3.5 ± 0.2 ng/mL; *P *= 0.031). When the 28 patients with different degrees of glomerulonephritis were excluded from the analysis, all measurements remained significantly different in SLE patients compared to controls, with the exception of sMerTK (not shown). From the rest of the manifestations, only thrombocytopenia showed significant differences in GAS6 values, increasing to 38.1 ± 3.5 ng/mL compared to 30.0 ± 1.5 ng/mL (*P *= 0.017), while signs of haemolytic anaemia increased MerTK values to 27.3 ± 3.7 ng/mL compared to 19.5 ± 1.6 ng/mL in patients without anaemia (*P *= 0.032).

### GAS6-TAM genetic variants and plasma concentrations in lupus and control patients

**Figure 3:**
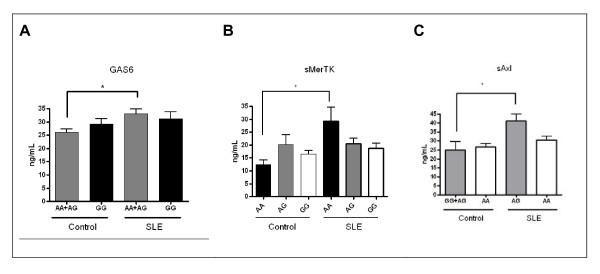
**Plasma concentrations of sMerTK, sAxl and growth arrest-specific gene 6 protein (GAS6) according to specific genotypes**. Genotypes giving significant differences in the concentration of their gene products are selected. (**A**) *GAS6 *rs8191974 (intron 8, c.834 ± 7G > A); (**B**) *MERTK *rs869016 (intron 1a, c.61 ± 2737G > A) and (**C**), *AXL *rs2304232 (intron 6 c.2169 ± 9A > G). SLE, systemic lupus erythematosus.

Next, we genotyped SNPs of the GAS6/ProS-TAM genes selected from a previously described population of patients with atherosclerotic disease and controls in the same geographical area [23,24]. From those determined, three SNPs showed clear differences among genotypes in their gene product plasma concentrations. For SNP rs869016 in *MERTK *intron 1 (c.61 ± 2737G > A), most of the difference in sMerTK concentration could be attributed to the AA group. The concentration of sMerTK increased from 12.3 ± 1.8 ng/mL in five AA controls to 29.3 ± 5.5 ng/mL in the eight AA homozygote patients. This concentration was significantly higher compared to the AG and GG patients (*P *= 0.019). Genotypes carrying the A allele of *GAS6 *intron 8 SNP variant (rs8191974) increased from 26.2 ± 1.3 ng/mL in 33 AA ± AG controls compared to 33.1 ± 1.8 ng/mL in 31 SLE patients (*P *= 0.002), while there was no difference in the GG group (Figure 3). In the case of *AXL*, the observed increase in sAxl could be attributed to carriers of the G allele (24.6 ± 5.2 ng/mL in 17 GG ± AG controls compared to 41.2 ± 3.8 ng/mL in 19 AG SLE patients), while no differences were observed in the AA genotype between SLE and controls. No differences in plasma concentrations were observed in relation to the other SNPs genotyped.

## Discussion

The GAS6/ProS-TAM system of vitamin K-dependent ligands and tyrosine kinase membrane receptors could have a prominent role in SLE, based in the observed phenotype of TAM knockout mice models [6,8]. Recent reports have evaluated the plasma concentrations of the soluble forms of the TAM receptors and ligands in SLE [18,20,22]. In the present study we measured all parameters in a population of SLE patients and compared them with a control population and with the activity of disease. The general pattern of variation of the GAS6/ProS-TAM system parameters in SLE is similar in the studies available, although we found a higher increase in sTyro3 and sAxl, while there was only a moderate increase in sMerTK plasma concentration, in contrast to a previous work [20]. The concentrations of the three receptors were in the ranges reported in most previously published studies, with sTyro3 < sMerTK < sAxl. Remarkably, both ligands, the vitamin K-dependent ProS and GAS6, were significantly different in the control and SLE populations, with higher GAS6 concentration and lower ProS in SLE patients. Previous results found a negative correlation between disease and free ProS, but no significant decrease [18]. GAS6 was found to be increased and correlated with SLEDAI in two studies [22,32], but not in a smaller sample [18]. Most likely, the differences among studies are due to the intrinsic heterogeneity of the populations in each case. Some studies have indicated that specific traits of SLE evaluated in SLEDAI, such as nephritis [20,22] or neurologic disorder [18], affect the values of GAS6 or sMerTK. We confirmed higher values of both GAS6 and sMerTK in patients with renal failure. In accordance with these findings, higher values of GAS6 have been reported in chronic renal failure [33]. The sMerTK values seem linked to kidney disease, as the increase was mainly in patients with renal failure. This was not the case for the other parameters.

A second source of heterogeneity is the effect of specific genetic factors on the plasma concentrations of GAS6, sAxl and sMerTK. Different populations are likely to present differences in the genetic distribution of the alleles studied, and this could be reflected in the plasma concentrations of the proteins. Indeed certain GAS6 variants have shown considerable differences between populations from Europe and Asia [23,24,34,35]. If some of these variants would have functional consequences, this could be reflected in plasma concentrations of the gene products. Due to the design and sample size of the study, it was not our goal to analyze the SNP distribution in SLE compared to the healthy population. A previous study in a Korean SLE population found no association between the risk of SLE and several *MERTK *polymorphisms, but found an association with leuco- and/or lymphopenia in SLE patients [36]. A recent study of GAS6 SNPs showed linkage with traits of SLE, including vasculitis (rs1803628) and thrombocytopenia (rs8191974), suggesting that larger studies could find associations between these genes and the development of certain disease traits [37].

Several parameters of the GAS6/ProS-TAM system correlated with the activity of disease measured through the SLEDAI score in our study, in line with previous findings [18,20,22]. We found significant positive correlations of SLEDAI with GAS6, sMerTK and sTyro3, similar to Wu *et al. *[20], but contrary to Ekman *et al. *[22], the concentration of sAxl did not correlate with the SLEDAI, while levels were found to be increased in both studies. It is possible that sAxl is correlated only indirectly with the disease activity, and thus this correlation is lost in some populations. It is interesting to note that sMerTK was the only parameter of the system correlated with damage caused by the disease as measured by SLICC.

Although the mechanism and significance underlying the observed differences in SLE patients cannot be inferred from the studies published until now, it is interesting to note that while GAS6 follows the soluble TAM receptors, augmenting SLE, ProS does not. GAS6 forms a stable complex with sAxl in human serum, where sAxl molar concentration is in excess of GAS6, blocking the activity of the ligand [38]. Furthermore, it has been shown that the presence of GAS6 activates the recycling of Axl receptors in the cell membrane [39]. Therefore, the increase observed in sTyro3, sAxl and GAS6 could reflect an increase in TAM regulatory activity due to the inflammation derived from the illness [40]. In this context, it is interesting to note that inflammatory molecules such as bacterial lipopolysaccharide induce TAM shedding [41]. The role of GAS6 activation of Axl and MerTK has been shown to be down-regulatory of the inflammatory innate response for several of its cellular components, including dendritic cells, natural killer cells and macrophages [40].

A second mechanism that could be reflecting the association of GAS6/ProS-TAM measurements and SLE is efferocytosis. Apoptotic cells, if not cleared properly, could release cellular contents that become self-antigens and develop the autoimmune reactions characteristic of pathologies such as SLE [4]. Presence of apoptotic cells in lymphatic tissue and decreased engulfment of apoptotic cells by macrophages and dendritic cells have been reported in SLE patients [42,43]. TAM kinases, and in particular MerTK, is essential in the proper clearance of apoptotic cells [40,44]. Some studies have suggested that the relevant MerTK ligand is ProS [45,46], but GAS6 has also been shown to activate MerTK [47]. ProS concentration is almost 1,000 times that of GAS6 in serum, and ProS has been shown to be the main serum adjuvant in efferocytosis [48]. The decrease of ProS detected in SLE patients is not large enough to be diagnosed as acquired ProS deficiency, and therefore, it is unlikely to have a strong effect on the risk of thrombosis. Still, it could have important consequences in the case of apoptotic cell clearance, which could be accentuated in SLE macrophages and dendritic cells [42,43,49].

## Conclusions

Here we show the association of several parameters related to the activity of TAM receptors and their ligands in patients with SLE. As suggested by other reports, GAS6 and other components of the system could be good markers of the development of SLE. Further studies with larger populations are needed in order to validate this assumption, as well as to determine the possible genetic components that could be best linked to the disease.

## Abbreviations

ANOVA: analysis of variance; C4BP: C4b-binding protein; ELISA: enzyme-linked immunosorbent assay; GAS6: growth arrest-specific gene 6 protein; Ig: immunoglobin; kDa: kiloDaltons; MAF: minor allele frequency; PBS: phosphate-buffered saline; PCR: polymerase chain reaction; ProS: protein S; SLE: systemic lupus erythematosus; SLEDAI: Systemic Lupus Erythematosus Disease Activity Index; SLICC: Systemic Lupus International Collaborating Clinics/American College of Rheumatology; SNPs: single nucleotide polymorphisms; TAM: Tyro3, Axl and MerTK receptor tyrosine kinases; VKDP: vitamin K-dependent glycoprotein.

## Competing interests

The authors declare that they have no competing interests.

## Authors' contributions

PRP executed experiments, and analysed the data. DT designed experiments supplied samples, clinical and laboratory data, and interpreted results. GE supplied samples and clinical data, and interpreted results. BH executed molecular genetic experiments and analysed the data. NS provided molecular genetic experimental resources and interpreted results. RC supplied samples and clinical data, and interpreted results. JCR conceived and designed experiments, supplied samples and clinical data, and interpreted results. PGDF conceived and designed the study, analysed and interpreted data and wrote the manuscript. All authors edited the manuscript, and read and approved the final version of the manuscript.
